# Salmonid Rickettsial Septicemia (SRS) disease dynamics and Atlantic salmon immune response to *Piscirickettsia salmonis* LF-89 and EM-90 co-infection

**DOI:** 10.1186/s13567-024-01356-0

**Published:** 2024-08-16

**Authors:** Gabriela Carril, Byron Morales-Lange, Marie Løvoll, Makoto Inami, Hanne C. Winther-Larsen, Margareth Øverland, Henning Sørum

**Affiliations:** 1 Department of Paraclinical Sciences, Faculty of Veterinary Medicine, Norwegian University of Life Sciences, 1432 Ås, Norway; 2 Department of Animal and Aquacultural Sciences, Faculty of Biosciences, Norwegian University of Life Sciences, 1433 Ås, Norway; 3 VESO Aqualab, 7810 Namsos, Norway; 4 Department of Pharmacology and Pharmaceutical Biosciences, Department of Pharmacy, University of Oslo, 0316 Oslo, Norway

**Keywords:** *Salmo salar*, Piscirickettsiosis, bacterial load, pathogenicity, immune-related biomarkers, bacterial intracellular pathogen, aquaculture, Co-culture

## Abstract

In Chile, *Piscirickettsia salmonis* contains two genetically isolated genogroups, LF-89 and EM-90. However, the impact of a potential co-infection with these two variants on Salmonid Rickettsial Septicemia (SRS) in Atlantic salmon (*Salmo salar*) remains largely unexplored. In our study, we evaluated the effect of *P. salmonis* LF-89-like and EM-90-like co-infection on post-smolt Atlantic salmon after an intraperitoneal challenge to compare changes in disease dynamics and host immune response. Co-infected fish had a significantly lower survival rate (24.1%) at 21 days post-challenge (dpc), compared with EM-90-like single-infected fish (40.3%). In contrast, all the LF-89-like single-infected fish survived. In addition, co-infected fish presented a higher presence of clinical lesions than any of the single-infected fish. The gene expression of salmon immune-related biomarkers evaluated in the head kidney, spleen, and liver showed that the EM-90-like isolate and the co-infection induced the up-regulation of cytokines (e.g., *il-1β*, *ifnγ*, *il8*, * il10*), antimicrobial peptides (*hepdicin*) and pattern recognition receptors (PRRs), such as *TLR5s*. Furthermore, in serum samples from EM-90-like and co-infected fish, an increase in the total IgM level was observed. Interestingly, specific IgM against *P. salmonis* showed greater detection of EM-90-like antigens in LF-89-like infected fish serum (cross-reaction). These data provide evidence that *P. salmonis* LF-89-like and EM-90-like interactions can modulate SRS disease dynamics in Atlantic salmon, causing a synergistic effect that increases the severity of the disease and the mortality rate of the fish. Overall, this study contributes to achieving a better understanding of *P. salmonis* population dynamics.

## Introduction

*Piscirickettsia salmonis* is a facultative intracellular Gamma-proteobacteria and the biological agent of the Salmonid Rickettsial Septicemia (SRS) or Piscirickettsiosis, a disease that causes significant economic losses in the Chilean salmon industry [1]. Nevertheless, this bacterium has also been reported in other major salmonid-producing countries (e.g., Scotland [2], Ireland [3], and Norway [4]), where it is considered an emerging fish disease, but has less impact on morbidity and mortality [5].

SRS leads to increased mortality of fish species such as Atlantic salmon (*Salmo salar*), Rainbow trout (*Oncorhynchus mykiss*), and Coho salmon (*Oncorhynchus kisutch*) by a systemic infection that predominantly affects the liver, kidney, and spleen [6]. Clinical signs (e.g., fish lethargy, pale gills, skin ulcers, and petechial hemorrhages [7]) are observed a few weeks after the transfer of smolts to seawater [8]. At the cellular level, *P. salmonis* infects and replicates within the cytoplasmic vacuoles of macrophages, which promotes an anti-inflammatory milieu for bacterial survival [8] and prevents lysosomal degradation [9] via the Dot/Icm Type IV-B Secretion System [10]. This induces high fish mortality rates, which, as an indicator of welfare, points to the poor overall health of infected farmed salmonids [11]. In addition, although vaccines are available against *P. salmonis*, these have been unsuccessful in preventing fish mortality caused by SRS. Thus, this disease is mainly controlled through the intensive use of antimicrobial agents [12, 13].

In Chile, the first *P. salmonis* outbreak was reported in Coho salmon in 1989 with the LF-89 strain (ATCC VR-1361) [14]. In 1990, a genetically diverged strain called EM-90 was described in Atlantic salmon [15]. These two strains were later used to classify *P. salmonis* isolates into genogroups due to genetic variability as indicative of virulence differences [16]. However, through exhaustive genomic analyses, it has recently been proposed that the genus *Piscirickettsia* consists of three genetically isolated genogroups [17]: LF-89, EM-90, and the Scottish, Norwegian, and Canadian isolates, which cluster together [18, 19]. Thus, the intergenogroup differences in pathogenesis are an important line of research for virulence factors related to the infection process [20], phylogenetic relationships among isolates [21], and genotypic background for epidemiology studies [22].

There are strategies to identify the two Chilean genogroups using different experimental approaches [23, 24]. For instance, by using specific probes for qPCR, the first evidence of co-infection by LF-89-like and EM-90-like genogroups in farmed Atlantic salmon was reported [25]. These findings indicate that both genogroups are co-localized at the same time, at the tissue and fish levels. Furthermore, co-culture of LF-89-like and EM-90-like isolates was found to induce changes in growth and biofilm production during in vitro analyses [26]. Additionally, evidence of differential expression of virulence factors triggered by in vivo co-culturing was presented. This indicates a synergistic effect in cohabitation that could be related to increased pathogenicity to the host during co-infection [26].

In fish, bacterial co-infections modulate the disease dynamics due to interactions between pathogens [27, 28], which may result in increased mortality rates linked to increased virulence via synergistic effects [29]. Likewise, immune responses can be affected through a cross-reactive response to different antigenic epitopes [30]. Related to SRS, many of the outbreaks caused by *P. salmonis* co-infection may have been undetected due to the diagnostic methods where culturing the bacterium from the field is needed, selecting for the most prevalent strain. Moreover, genotyping is not required by the official surveillance program [25]. Therefore, evaluating whether co-infection affects the development of the disease and its relationship with salmonid mortality is relevant for fish farming.

Our study aimed to assess the co-infection of Atlantic salmon with *P. salmonis* LF-89-like and EM-90-like isolates by comparing their pathogenicity and disease dynamics to determine whether the bacterial interaction led to potential changes in virulence associated with the fish immune response and mortality. This may contribute to the development of new effective control strategies through the improvement of the disease model used to study SRS and a better understanding of *P. salmonis* population dynamics.

## Materials and methods

### Fish

Atlantic salmon (StofnFiskur strain) were reared at VESO Aqualab Hatchery (Fosslandsosen, Norway). Before the fish trial started, all the fish were tested by ELISA for specific antibody activity in plasma (against *Vibrio salmonicida*, *Vibrio anguillarum* O1 and O2a, *Vibrio ordalii*, *Aeromonas salmonicida*, *Moritella viscosa*, *Yersinia ruckeri*, and infectious pancreatic necrosis virus (IPNV)) and screened by qPCR for infectious salmon anaemia virus (ISAV), salmon pancreas disease virus (SPDV), piscine orthoreovirus (PRV) and infectious pancreas necrosis virus (IPNV). All the fish were negative for the analysed pathogens. Then, 252 unvaccinated Atlantic salmon (average weight: 60.4 g) were smoltified by light manipulation. The fish were exposed to 12 h of light and 12 h of darkness (12:12) for 6 weeks before being transferred to the experimental test facility at VESO Aqualab (Namsos, Norway) to brackish water (25‰ ± 2‰, 15 °C) with continuous 24 h of light exposure (24:0).

### *Piscirickettsia salmonis* culture

Two *P. salmonis* isolates were used for single and co-infections, Psal-013 from the LF-89 genogroup and Psal-182 from the EM-90 genogroup [26]. A standard procedure involving the culture of bacteria from cryovials stored at − 80 °C in FN2 broth medium [29] was followed. Briefly, 100 µL of culture was plated on cysteine heart agar (CHAB) supplemented with ovine blood (5%) and incubated at 18 °C for ten days. Thereafter, one single colony was grown in FN2 broth medium with agitation (100 rpm) at 18 °C. To measure the density of the liquid culture used to prepare the inoculum for the challenge, a Jenway 6300 spectrophotometer was used, and the cultures were adjusted following the protocol in Meza et al. [31].

### Bacterial challenge

The fish were starved for 48 h before the challenge and divided into three groups (80 fish each) in three identical tanks with a stocking density of 40 kg/m^3^. During the trial, the fish were fed ad libitum with a commercial diet (Skretting AS) and monitored daily. To perform the challenge, the fish were sedated using AQUI-S VET (isoeugenol, MSD Animal Health) and intraperitoneally (i.p.) injected with 0.1 mL of different *P. salmonis* strains at a 1:1 ratio for co-infections (Table 1). This was carried out according to the i.p. challenge model described by Meza et al. [31]. Fish at the terminal stage with clear signs of disease (erratic swimming, lethargy, pale gills, and ulcers in the skin) were euthanized with an overdose of benzocaine chloride (Benzoak 200 mg/mL) and recorded as mortality. At 0 days (as a negative control before the challenge) and 7, 14, and 21 days post-challenge (dpc), 12 fish were randomly selected, euthanized (previously described) and sampled for head kidney, liver, and spleen collection in RNA Later  (R0901, Sigma‒Aldrich). In addition, blood samples were collected to obtain serum by centrifugation (800 × *g*) for 10 min at 4 °C.

**Table 1:** **Bacterial challenge (by intraperitoneal injection) of Atlantic salmon post-smolts with theoretical doses of** ***P. salmonis.***

*P. salmonis* strains	Dose (cfu/mL)	Volume (dose/fish)	No. fish
Psal-013 (LF-89-like)	1.0 × 10^7^	0.1 mL	80
Psal-182 (EM-90-like)	1.0 × 10^7^	0.1 mL	80
Psal-013 (LF-89) + Psal-182 (EM-90)	1.0 × 10^7^ + 1.0 × 10^7^	0.05 mL + 0.05 mL	80

### RNA extraction

For total RNA extraction, samples were weighed (10 mg for spleen and 20 mg for head kidney and liver) and homogenized using 5 mm stainless steel beads (Qiagen, Hilden, Germany) in a TissueLyser II (Qiagen) for 30 s at 30 Hz. Then, the RNeasy Mini Kit (Qiagen) was used for RNA extraction according to the manufacturer’s protocol. The concentration and quality were measured using a Multiskan Sky Microplate Spectrophotometer (Thermo Fisher Scientific, Waltham, MA, USA). RNA samples were stored at − 80 °C until use.

### DNA extraction

For DNA extraction, a pool composed of an equal quantity (10 mg) of tissue per sample point for each experimental group was made (following Martínez et al. [32]). These 27 samples were homogenized using 5 mm stainless steel beads (Qiagen) in a TissueLyser II (Qiagen) for 30 s at 30 Hz. Then, the samples were incubated overnight with protein kinase at 56 °C, and the QIAGEN DNeasy Blood and Tissue Kit (Qiagen) was used according to the manufacturer’s protocol. The concentration and quality of the DNA obtained were measured using a Multiskan Sky Microplate Spectrophotometer (Thermo Fisher Scientific), and the DNA was stored at − 80 °C until use.

### RT-qPCR analysis

RNA samples were used for cDNA synthesis with a QuantiTect Reverse Transcription Kit (Qiagen) according to the manufacturer’s protocol. Immune-related genes were evaluated (Table 2) using an Agilent AriaMx Real-Time PCR system (Agilent Technologies, Santa Clara, CA, USA). Each reaction included 10 µL of PowerUp SYBR Green Master Mix (Thermo Fisher Scientific), 0.3 µM of each primer, and 15 ng of cDNA template in a final volume of 20 µL. All samples were tested in triplicate for each target gene. The thermal cycling conditions were as follows: 2 min at 50 °C for UDG pretreatment, an initial denaturation of 5 min at 95 °C and 40 cycles of 15 s at 95 °C, 30 s at 60 °C for annealing and 30 s at 72 °C for extension before a melting curve was obtained. Ct values were normalized to the relative expression of *ef1α* and transformed to the 2^−ΔΔCt^ method [33].

**Table 2:** **List of primers used for qPCR.**

Gene or bacterial genogroups	Organism	Primers (5′–3′)	NCBI reference sequence
*ef-1α* *Elongation factor 1-alpha*	*S. salar*	F: CCCCTCCAGGACGTTTACAAA R: CTAAACGAAGCCTGGCTGTAAACG	NM_001123629.1
*il-1β* *Interleukin 1 beta*	*S. salar*	F: ATCACCATGCGTCACATTGC R: GTCCTTGAACTCGGTTCCCA	NM_001123582.1
*il-8* *Interleukin 8*	*S. salar*	F: GGCCCTCCTGACCATTACT R: ATGAGTCTACCAATTCGTCTGC	NM_001140710.3
*il-10* *Interleukin 10*	*S. salar*	F: ACAACAGAACGCAGAACAACC R: GCATAGGACGATCTCTTTCTTCAG	XM_045705802.1
*tnfα* *Tumor necrosis factor alpha*	*S. salar*	F: GCAGCCATCCATTTAGAGGGTGAA R: CTAAACGAAGCCTGGCTGTAAACG	NM_001123589.1
*ifnγ* *Interferon gamma*	*S. salar*	F: CTAAAGAAGGACAACCGCA R: CACCGTTAGAGGGAGAAATG	NM_001171804.1
*Hepcidin*	*S. salar*	F: TGTTCCTTTCTCCGAGGT R: AAAGCCACAGCCAATGT	XM_014170058.2
*tlr5s* *Toll-like receptor 5*	*S. salar*	F: GCTGCTGGAGCTAAGGAACA R: GAGCCCTCAGCGAGTTAAGC	HQ664668.1
*glyA* *Serine hydroxymethyltransferase*	*P. salmonis*	F: CGCGTACCATTGCAGATTTCGACC R: GCTTCTAGCACACGCGGACTCG	QGP40124.1
LF13	*P. salmonis*	F: AAAGAGCCCTGACCAAACAA R: CCCTGAGTTGTCAACAGCAA	QGO18456.1
EM182	*P. salmonis*	F: CTCTACGCATGGGAACAGTG R: CACCACCAACAACACTACCG	QGP37999.1

*F* forward, *R* reverse, *NCBI* National Center for Biotechnology Information.

### Detection of bacterial load

To quantify the bacterial load in the tissue samples, total DNA was used as a template for the qPCR analyses. Threshold cycle (Ct) values were used as an indication of the bacterial load. A single copy of the *glyA* gene was used as a marker for bacterial replication during infection (primers are listed in Table 2), along with specific primers for each genogroup, as described previously [26] (Table 2). qPCR was performed using an Agilent AriaMx Real-Time PCR system and PowerUp SYBR Green Master Mix (Thermo Fisher Scientific) with a reaction mixture of 0.3 µM for each primer and 15 ng of DNA template in a final volume of 20 µL. The qPCR protocol was as follows: 2 min at 50 °C, 5 min at 95 °C and 40 cycles of 15 s at 95 °C, 30 s at 60 °C and 30 s at 72 °C, followed by melting curve analysis. All tissues sampled at each time point were tested in triplicate for each target gene with all primer sets.

### Necropsy

Using 12 fish per challenge group sampled at 14 and 21 dpc, macroscopic lesions were analysed [34]. The pathological signs included the presence or absence of ascites, pale nodules in the liver, swollen liver, swollen spleen, intestinal bleeding, distended ventricle, and general hemorrhages.

### Enzyme-linked immunosorbent assay (ELISA)

Serum samples from 0, 14, and 21 dpc were analysed by ELISA to determine total and specific IgM (against *P. salmonis*) according to Figueroa et al. [12]. First, in each serum sample, total proteins were quantified by the BCA Protein Assay Kit (Thermo Fisher Scientific) following the manufacturer’s instructions. Then, for total IgM, the serum samples were diluted (50 ng µL^−1^, 100 µL) in bicarbonate buffer (sodium bicarbonate, 60 mM, pH 9.6) and seeded in duplicate on Nunc Maxisorp plates (Thermo Fisher Scientific). After overnight incubation at 4 °C, the plates were washed 3 times with PBS-T (PBS with Tween-20 at 0.2%) and incubated with blocking solution (200 µL per well of Clear Milk Blocking Buffer 1x, Bio-Rad, Hercules, CA, USA) for 2 h at 37 °C. The plates were again washed 3 times with PBS-T and incubated (100 µL per well) with a primary antibody (monoclonal anti-salmonid IgM, Ango #FM-190AZ-5) for 90 min at 37 °C. The primary antibody was washed with PBS-T (3 times), and the plates were incubated (100 µL per well, 60 min at 37 °C) with the secondary antibody (goat anti-mouse IgG, HRP-conjugated) from Thermo Fisher Scientific (#31430). Finally, the secondary antibody was also washed with PBS-T, and the plates were incubated with tetramethylbenzidine (TMB) single solution (Thermo Fisher Scientific) for 10 min (in the dark) at room temperature (100 µL per well). All reactions were stopped with 50 μL of sulfuric acid (1 N), and the plates were read at 450 nm on a SpectraMax microplate reader (Molecular Devices, San Jose, CA, USA). In parallel to the serum samples, a standard of plasma immunoglobulins from Atlantic salmon was used to quantify total IgM in plasma.

For the detection of specific IgM against *P. salmonis*, total proteins from each bacterial genogroup were extracted from 200 mL of liquid culture in the exponential phase. The cultures were centrifuged (4000×*g* for 15 min at 4 °C), and the supernatant was removed. Then, the bacteria were inactivated for 10 min at 70 °C and quickly placed on ice, after which 5 mL of RIPA lysis buffer (Thermo Fisher Scientific) supplemented with cOmplete Protease Inhibitor (Sigma‒Aldrich) was added. These solutions were sonicated and centrifuged at 10,000 × *g* for 20 min at 4 °C, after which the supernatant was recovered. Total proteins in the supernatant were quantified with a BCA protein assay kit (Thermo Fisher Scientific). Thereafter, the proteins from the LF-89-like isolate (Psal-013), the EM-90-like isolate (Psal-182), and a mixture of both (1:1 ratio) were seeded at 50 ng µL^–1^ (100 µL per well) and incubated overnight (at 4 °C) on Nunc Maxisorp plates. Similar to the protocol described above, the plates were washed and incubated with a blocking solution. Afterwards, 70 ng µL^−1^ (100 µL) of total IgM from each serum sample was incubated in duplicate (90 min at 15 °C) in each of the plates with the different antigens. Following this, the ELISA protocol (mouse anti-IgM antibody, goat anti-mouse IgG HRP-conjugated, and TMB) was the same as that previously used.

### Statistical analyses

The data were analysed, and graphs were generated using GraphPad Prism (v8.0.1). Survival analysis was performed with a survival curve based on the Kaplan‒Meier method, while the Log-rank test was used to compare survival curves. Moreover, differences in the clinical signs of *P. salmonis* infection between different groups were analysed using a non-parametric Chi-square test. The RT-qPCR results were presented as means and were checked for normality (Shapiro–Wilk test) and then log_2_-transformed [35] before being analysed by one-way ANOVA and Tukey’s multiple comparisons test. ELISA data were also analysed using ANOVA followed by Tukey’s multiple comparisons test. All differences were considered significant when the *p* value was < 0.05.

## Results

### *P. salmonis* co-infection caused a significantly lower survival rate than single infections

**Figure 1:**
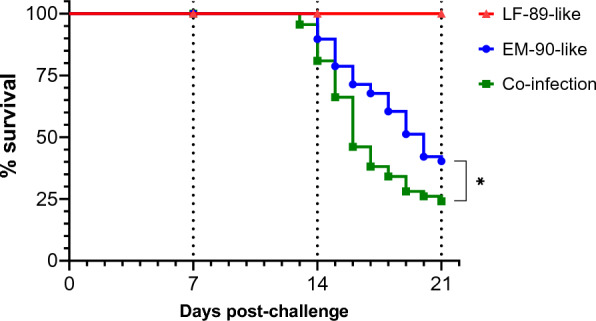
**Survival rate (in percentages) of post-smolt Atlantic salmon i.p. challenged with *P. salmonis.*** Vertical dashed lines: sampling days. Triangles: LF-89-like isolate (Psal-013). Circles: *P. salmonis* EM-90-like isolate (Psal-182). Squares: Co-infection with both isolates (ratio 1:1). *n* = 80 fish per group. dpc: Days post-challenge. *: significant difference (*p* value < 0.0001).

The survival rates of single- and co-infected Atlantic salmon with the LF-89-like and EM-90-like isolates are shown in Figure 1. During infection with the LF-89-like isolate (Psal-013), the fish showed a 100% survival rate, while infection with the EM-90-like isolate (Psal-182) resulted in a survival rate of 40.3% within 21 dpc. However, when the isolates were mixed for the co-infection challenge, the survival rate of the fish decreased to 24.1% after 21 dpc, and since mortality started one day earlier at 13 dpc, the survival curve had a steeper slope. Moreover, a significant difference (*p* value < 0.0001) between survival curves was detected.

### Increased pathological changes during co-infection challenge

**Figure 2:**
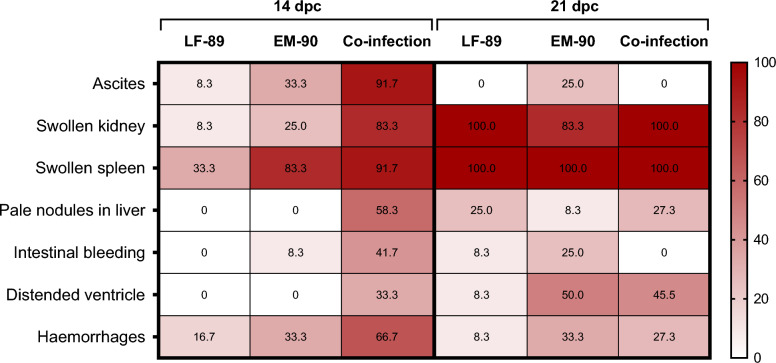
**Pathological changes observed in post-smolt Atlantic salmon i.p. challenged with *P. salmonis* LF-89-like isolate (Psal-013), EM-90-like isolate (Psal-182) or after co-infection with both isolates (ratio 1:1).** The intensity of colour shows the frequency as a percentage (%) of the sampled fish with the pathological signs listed at 14 and 21 days post-challenge (dpc). 100% corresponds to *n* = 12.

During the infection experiment, pathological changes in the fish were monitored. The presence or absence of pathological changes is shown in Figure 2. In general, a higher presence of clinical signs was observed in the co-infection challenge group after 14 dpc. For instance, compared to the EM-90-like group, the co-infected fish had a significantly higher incidence of ascites, pale nodules in the liver, a swollen kidney, and a distended ventricle (Table 3). Furthermore, the difference in incidence between LF-89-like single infection and co-infection was significant for all clinical signs.

**Table 3:** **Data analysis of clinical signs was performed by the Chi-square test for each experimental comparison.**

Clinical sign	Comparison	Chi-square		df		*p*-value	
		14 dpc	21 dpc	14 dpc	21 dpc	14 dpc	21 dpc
Ascites	LF-89|EM-90	8.71	3.43	1	1	0.003*	0.06
	LF-89|Co-infection	16.70	n/a	1	n/a	< 0.0001*	n/a
	EM-90|Co-infection	8.71	3.43	1	1	0.003*	0.06
Swollen kidney	LF-89|EM-90	1.20	2.18	1	1	0.27	0.14
	LF-89|Co-infection	13.60	1.04	1	1	0.0002*	0.31
	EM-90|Co-infection	8.22	0.38	1	1	0.004*	0.54
Swollen spleen	LF-89|EM-90	6.17	n/a	1	n/a	0.01*	n/a
	LF-89|Co-infection	8.71	1.04	1	1	0.003*	0.31
	EM-90|Co-infection	0.38	1.04	1	1	0.54	0.31
Pale nodules in the liver	LF-89|EM-90	n/a	1.20	n/a	1	n/a	0.27
	LF-89|Co-infection	9.88	0.00	1	1	0.002*	> 0.99
	EM-90|Co-infection	9.88	1.20	1	1	0.002*	0.27
Intestinal bleeding	LF-89|EM-90	1.04	1.20	1	1	0.31	0.27
	LF-89|Co-infection	6.32	1.04	1	1	0.01*	0.31
	EM-90|Co-infection	3.56	3.43	1	1	0.06	0.06
Distended ventricle	LF-89|EM-90	n/a	5.04	n/a	1	n/a	0.02*
	LF-89|Co-infection	4.80	3.56	1	1	0.03*	0.06
	EM-90|Co-infection	4.80	0.17	1	1	0.03*	0.68
Haemorrhage	LF-89|EM-90	0.68	2.27	1	1	0.41	0.1316
	LF-89|Co-infection	6.17	1.20	1	1	0.01*	0.2733
	EM-90|Co-infection	2.67	0.20	1	1	0.10	0.6534

* significant difference (p-value < 0.05)

*df* degree of freedom, *dpc* days post-challenge

### Differential bacterial loads

**Figure 3:**
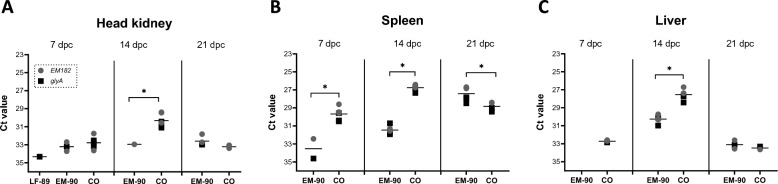
***P. salmonis* load measured by qPCR in DNA samples from the head kidney (A), spleen (B), and liver (C) of post-smolt Atlantic salmon i.p. challenged with *P. salmonis* LF-89-like isolate (Psal-013), EM-90-like isolate (Psal-182) or after co-infection with both isolates (CO).** The black square indicates the single-copy gene *glyA* from *P. salmonis*. The grey circle indicates the unique EM-90 gene, which was amplified with custom-designed primers (EM182). Values for LF-89-specific primers are not shown due to detection below the threshold for all samples. Ct values are presented as the means. Days post-challenge: dpc. *Significant difference (*p* value < 0.05).

The bacterial loads of the *P. salmonis* LF-89-like and EM-90-like isolates during the co-infection experiments were estimated using unique genes for each genotype via DNA in samples collected during the i.p. challenge, in addition to total detection with *glyA* (Figure 3). In head kidney samples, LF-89-like was detected at 7 dpc (without a significant difference from EM-90-like or co-infected fish), but a significantly greater load of *P. salmonis* was detected at 14 dpc in co-infected fish compared to EM-90-like infected fish (Figure 3A). Moreover, a significantly greater number of *P. salmonis* was detected in co-infected fish compared to EM-90-like-infected fish (at 7 and 14 dpc in the spleen). However, at 21 dpc, this profile changed, and significantly less *P. salmonis* was detected in co-infected fish compared to EM-90-like-infected fish (Figure 3B).

In the liver, *P. salmonis* was detected only in the co-infected group at 7 dpc, and this group reached the significantly highest concentration of *P. salmonis* compared to that in the EM-90-like-infected fish at 14 dpc (Figure 3C).

### Gene expression of immune-related biomarkers in Atlantic salmon

**Figure 4:**
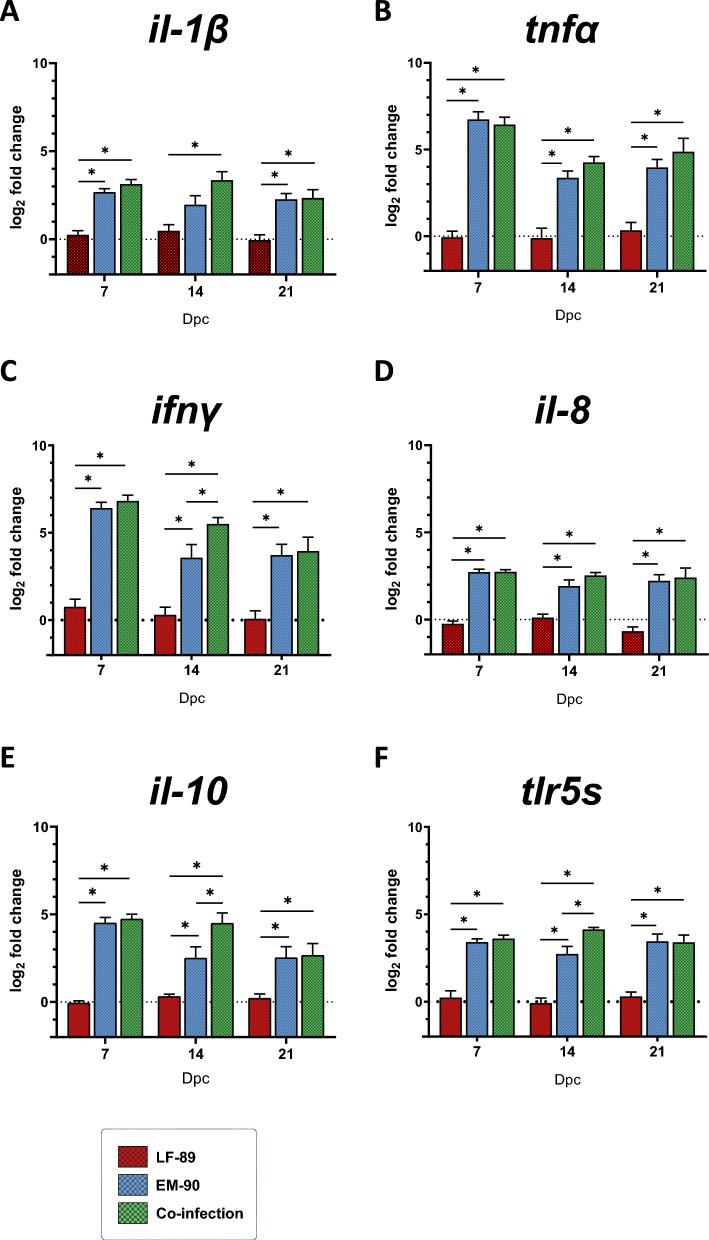
**Gene expression (by RT-qPCR) of immune-related biomarkers in head kidney samples from post-smolt Atlantic salmon i.p. challenged with *P. salmonis* LF-89-like isolate (Psal-013) or EM-90-like isolate (Psal-182) or after co-infection with both isolates (ratio 1:1).** The data are displayed in log_2_-fold change compared to the control group (0 days post-challenge). A: *il-1β.* B: *tnfα.* C: *ifnγ*. D: *il-8*. E: *il-10*. F: *tlr5s*. dpc: days post-challenge. *Significant difference (*p* value < 0.05). The error bars indicate the mean ± SEM.

**Figure 5:**
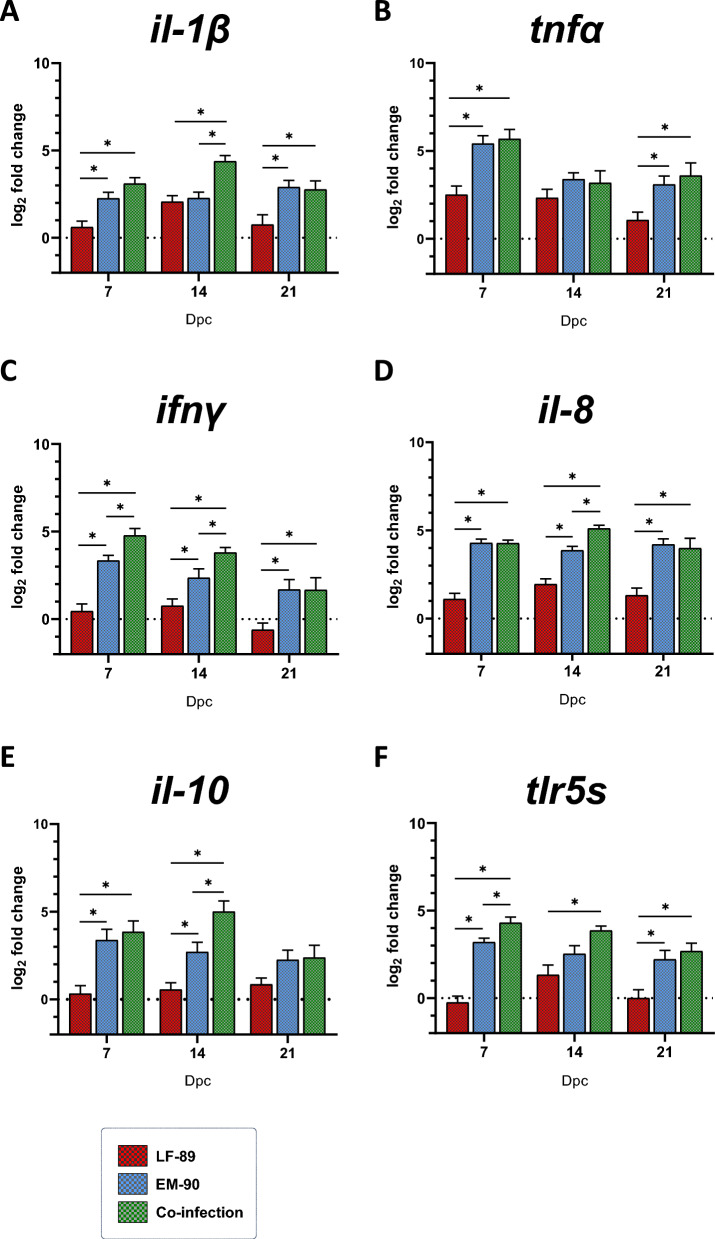
**Gene expression (by RT-qPCR) of immune-related biomarkers in spleen samples from post-smolt Atlantic salmon i.p. challenged with *P. salmonis* LF-89-like isolate (Psal-013) or EM-90-like isolate (Psal-182) or after co-infection with both isolates (ratio 1:1).** The data are displayed in log_2_-fold change compared to the control group (0 days post-challenge). A: *il-1β.* B: *tnfα.* C: *ifnγ*. D: *il-8*. E: *il-10*. F: *tlr5s*. dpc: days post-challenge. *Significant difference (*p* value < 0.05). The error bars indicate the mean ± SEM.

**Figure 6:**
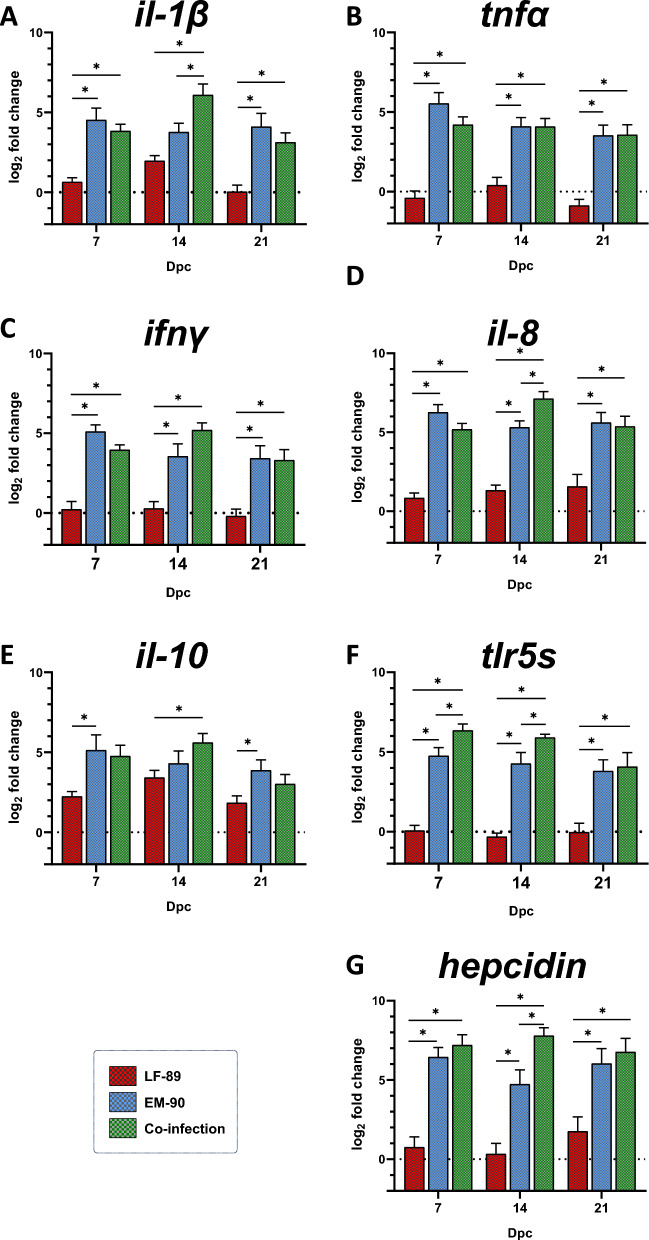
**Gene expression (by RT-qPCR) of immune-related biomarkers in liver samples from post-smolt Atlantic salmon i.p. challenged with *P. salmonis* LF-89-like isolate (Psal-013) or EM-90-like isolate (Psal-182) or after co-infection with both isolates (ratio 1:1).** The data are displayed in log_2_-fold change compared to the control group (0 days post-challenge). A: *il-1β.* B: *tnfα.* C: *ifnγ*. D: *il-8*. E: *il-10*. F: *tlr5s*. G: *hepcidin.* dpc: days post-challenge. *Significant difference (*p* value < 0.05). The error bars indicate the mean ± SEM.

To gain a better understanding of the effects of LF-89-like/EM-90-like co-infection on the host immune response, gene expression analysis of immune-related biomarkers was performed on fish immune organs such as the head kidney, spleen, and liver (Figures 4, 5, and 6). Compared with both the EM-90-like-infected and co-infected fish, the LF-89-like-single-infected fish exhibited the lowest expression pattern of each immune gene evaluated (Figures 4, 5, and 6), in concordance with the observed zero mortality (Figure 1).

In terms of pro-inflammatory cytokines, *il-1β* in the spleen and liver was up-regulated in co-infected fish compared to EM-90-like-infected fish at 14 dpc (Figures 5A and 6A). In addition, although the expression of *tnfα* peaked at 7 dpc in the head kidney, spleen, and liver, it showed a different pattern of up-regulation compared to that of the other cytokines evaluated, which diminished over time. No significant difference in the expression of *tnfα* was observed in EM-90-like-infected and co-infected fish (Figures 4B, 5B, and 6B). However, another pro-inflammatory cytokine, *ifnγ*, was significantly up-regulated between EM-90-like-infected fish and co-infected fish at 7 dpc in the spleen (Figure 5C) and at 14 dpc in the head kidney and spleen (Figures 4C and 6C).

The chemoattractant cytokine *il-8* was significantly up-regulated in the livers of co-infected fish compared to those of EM-90-like-infected fish (at 7 and 14 dpc) and at 14 dpc in the head kidney (Figures 5D and 6D).

The expression of the anti-inflammatory cytokine *il-10* in the head kidney and spleen was significantly increased at 14 dpc in co-infected fish compared to EM-90-like single-infected fish (Figures 4E, 5E). Moreover, there was a significant up-regulation of the soluble toll-like receptor (*tlr5s*) in co-infected fish compared to that in EM-90-like-infected fish at 7 dpc in the spleen and liver and at 14 dpc in the head kidney and liver (Figures 4F, 5F, and 6F).

Finally, the antimicrobial peptide *hepcidin* was only expressed in the liver and was significantly up-regulated in co-infected fish compared to EM-90-like-infected fish at 14 dpc (Figure 6G).

### Detection of immunoglobulins in serum

**Figure 7:**
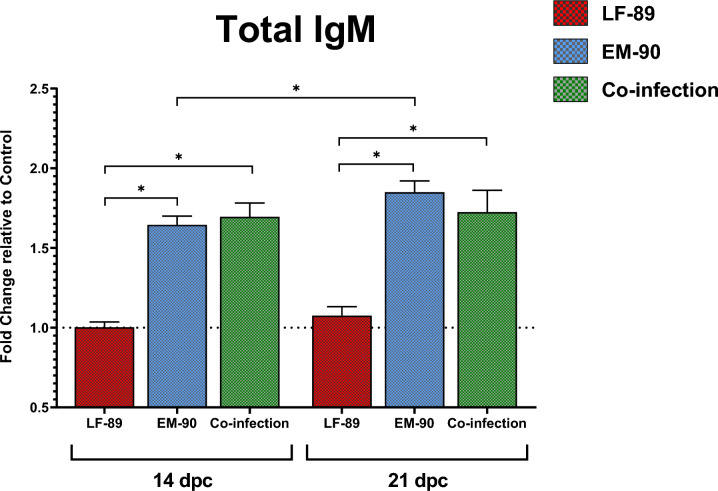
**Detection of total IgM (by ELISA) in serum samples from post-smolt Atlantic salmon i.p. challenged with *P. salmonis*.** LF-89-like isolate (Psal-013) is shown in red. In blue: EM-90-like isolate (Psal-182). Green: co-infection with both isolates (ratio 1:1). The data are expressed as the fold change relative to the control group (0 days post-challenge). dpc: days post-challenge. *Significant difference (*p* value < 0.05). The error bars indicate the mean ± SEM.

Significant differences in the level of total IgM between LF-89-like and EM-90-like-infected fish and between LF-89-like and co-infected fish were observed at 14 dpc and 21 dpc (Figure 7). Regarding sampling days, a significant increase in total IgM was observed between 14 and 21 dpc in EM-90-like infected fish.

**Figure 8:**
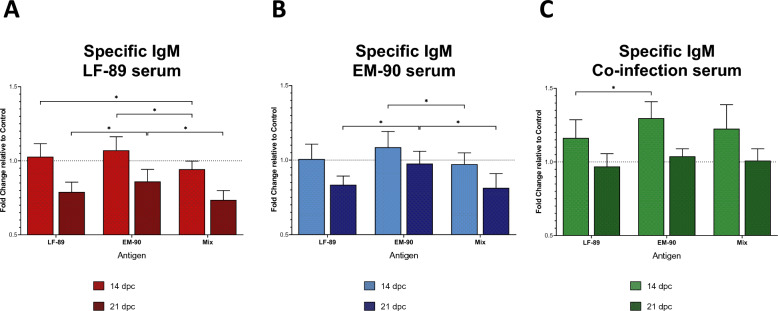
**IgM levels were measured (by ELISA) in serum samples from post-smolt Atlantic salmon i.p. challenged with *P. salmonis*.** LF-89-like isolate (Psal-013) is shown in red. In blue: EM-90-like isolate (Psal-182). Green: co-infection with both isolates (ratio 1:1). Three different antigens were used: LF-89-like (total proteins from Psal-013), EM-90-like (total proteins from Psal-182), and Mix (mixture of LF-89-like and EM-90-like antigens). The data are expressed as the fold change relative to the control group (0 days post-challenge). dpc: days post-challenge. *Significant difference (*p* value < 0.05). The error bars indicate the mean ± SEM.

The detection of specific IgM against *P. salmonis* analysis showed that compared with the antigen mixture, the LF-89-like-infected fish had significantly higher levels of plasma antibodies against EM-90-like and LF-89-like at 14 dpc (Figure 8A). However, the level of specific IgM against EM-90-like was significantly higher than that against both LF-89-like and the mixture of both at 21 dpc.

Furthermore, using EM-90-like infected fish serum (Figure 8B), the highest level of specific IgM was detected at 14 dpc against EM-90-like, which was significantly higher than that detected for the mixture of both genogroups but not for LF-89-like. In contrast, at 21 dpc, the level of specific IgM against EM-90-like was significantly higher than that against the antigen mixture and LF-89-like . Finally, using co-infected fish serum the amount of *P. salmonis-*specific IgM detected in response to EM-90-like antigen was significantly higher than that detected in response to LF-89-like at 14 dpc (Figure 8C).

## Discussion

In Chile, SRS caused by *P. salmonis* is the leading cause of salmonid mortality among infectious diseases [36]. Traditionally, research on this bacterium has predominantly investigated single infections or interactions with the ectoparasite *Caligus rogercresseyi*, which is responsible for caligidosis, another important sanitary challenge in the Chilean salmon industry [37, 38]. Overlooking the potential complexities arising from the concomitant presence of the LF-89 and EM-90 genogroups. This, in addition to the evidence of natural co-infection in farmed salmon, allows us to raise the question of whether *P. salmonis* co-infection could be one of the factors contributing to Chile’s higher fish mortality compared to other countries where this bacterium is present.

In the present study, we report the co-infection challenge of Atlantic salmon with *P. salmonis* LF-89-like and EM-90-like isolates, and the experimental results showed that SRS disease dynamics was modulated from the single- to the co-infection challenge since the host outcome presented a higher presence of clinical lesions and a lower survival rate after co-infection. This is in line with previous work in which fish infected with two closely related species of a bacterial pathogen (*Vibrio harveyi* and *Vibrio alginolyticus*) displayed more severe histological alterations and clinical symptoms [39].

Compared with those of EM-90-like single infection and co-infection, the effects of LF-89-like single infection were milder, with no mortality and fewer observed pathological changes. This was consistent with the lower detection of the LF-89-like isolate DNA. A possible explanation could be that LF-89-like cells were not viable at the time of i.p. injection. However, bacterial cultures (on agar plates) demonstrated the growth and viability of the same *P. salmonis* LF-89-like inoculum used for the i.p. injection (data not shown). Only the head kidney showed the presence of LF-89-like cells at 7 dpc, which could indicate the migration of the bacteria after intraperitoneal injection. Nevertheless, an early sampling time, such as 24 h post-challenge, could have been a better option for comparing successful infections among the groups. Rozas-Serri et al. [40], who used a cohabitation challenge model, reported that the abundance of LF-89-like isolates starts to increase after 28 dpc. Thus, our last sampling at 21 dpc may have been premature for bacterial detection using a qPCR strategy. Additionally, Rozas-Serri et al. [40] reported that fish with LF-89 infections seem to have less severe disease and lower mortality. This finding contrasts with that for EM-90-like infected fish, for which the results of the challenge were similar to those of previous studies and was related to higher mortality within a shorter time period compared to LF-89-infected fish [40–42]. Although the LF-89-like isolate was not infective in our study, the co-infection with EM-90-like induced more acute and faster disease development. Compared with the other single-challenged groups, co-infected fish reached a higher bacterial load at 14 dpc in the head kidney, spleen, and liver, as well as exhibited more pathological changes, a lower survival rate, and increased expression of host immune-related biomarkers. This was in accordance with the negative impact of infections by multiple pathogens described in fish, which can be associated with alterations in disease dynamics, increased severity, and evasion of the immune response [43].

The mechanism underlying the observed effect of co-infection remains unclear, but since the initial bacterial inoculum was theoretically identical for each challenged group, the differences are probably the result of interactions between the isolates. These data resemble the synergistic effect observed at in vitro level during LF-89-like and EM-90-like co-cultures, which promotes the expression of virulence factors that could worsen infection in Atlantic salmon [26]. However, it is important to note that the observed differences in survival rates could be because each treatment group was kept in a single tank due to tank space limitations, even though we used individual fish as the experimental unit of study for statistical analyses to overcome this limitation. In this regard, similar experimental designs have already been used to study SRS in Atlantic salmon [44, 45].

Clinical outcomes do not necessarily correspond to the establishment of an infection. Thus, evaluating and comparing the host immune response to single and co-infections may provide additional insights into *P. salmonis* pathogenic mechanisms. For instance, it has been proposed that during bacterial infection, one virulence mechanism is the expression of flagellin (protein monomer) [46], which functions as a ligand detected by the host through pattern recognition receptors (PRRs) [47]. The genes encoding the flagellar system, such as the hook-associated protein FlgK, are important virulence factors found in transcriptomic analyses of *P. salmonis* [26, 46, 48, 49]. Although this bacterium is described as non-motile, there is evidence of an active flagellar gene cluster that leads to the synthesis and secretion of a flagellin monomer that could be involved in the infection process and modulation of the host immune response [48]. Atlantic salmon has two TLR5-binding flagellins, TLR5 soluble, and membrane-localized TLR5 (TLR5S and TLR5M, respectively) [47, 50]*.* These receptors are related to the activation of a pro-inflammatory process [51, 52]. Using head kidney leukocytes (HKLs) from Atlantic salmon, studies on stimulation with flagellin showed that the expression of TLR5M at 3 h post-stimulation was only 0.3-fold higher, while that of TLR5S increased by 26-fold at 6 h post-stimulation [53]. In addition, NF-κβ activation is modulated by TLR5S, which then also induces a cellular response to flagellin mediated by TLR5M [54, 55]. This allowed us to consider TLR5S as an interesting biomarker to describe the overall response of TLR5 in Atlantic salmon. Consistently, our results showed that *tlr5s* was significantly up-regulated in co-infected fish compared to EM-90-like and LF-89-like single infections during the early stage of the infection process (at 7 dpc in the spleen and liver and at 14 dpc in the liver and head kidney), suggesting increased detection of flagellin from *P. salmonis* during co-infections.

In fish such as Orange-spotted grouper (*Epinephelus coioides*), the up-regulation of TLR5S also induced an increase in pro-inflammatory cytokines (e.g., *ifnγ*, *il-6*, and *tnfα*) [56]. In Atlantic salmon, these types of cytokines have been reported to be modulated in previous challenge models (i.p. injection and cohabitation) with *P. salmonis* EM-90-like isolates, suggesting a modulation of the pro-inflammatory response [42, 57]. Moreover, similar to our data, it has been described that *il-1β* and *il-8* can be up-regulated during in vitro infections of SHK-1 cells (salmon head kidney cells) with planktonic EM-90 [58].

While IL-1β is a pro-inflammatory cytokine produced after PRRs are activated by pathogens or danger-associated molecular patterns (PAMPs or DAMPs) [59], and it affects the phagocytic and lysosomal activity of macrophages [60] for antibacterial defense [61]*,* IL-8 is a member of the CXC chemokine family whose biological function is to recruit leukocytes to infection sites [62]. Moreover, IL-8 can induce IFNγ expression, triggering a signalling cascade [59]. This finding is interesting since TLR5S can also lead to the modulation of IFN-mediated responses [63], and our data are consistent with this idea since, in this study, *ifnγ* was up-regulated during co-infection compared with single infections at earlier time points, which agreed with previous transcriptome analysis of *P. salmonis* infection in Atlantic salmon [57].

In teleost fish, IFNγ promotes the activation of M1 macrophages related to pro-inflammatory processes [64]. For instance, IFNγ can improve phagocytosis and enhance the production of reactive species in addition to modulating other cytokines, such as TNFα [65]. In addition, after stimulation with IFNγ, Atlantic salmon antigen-presenting cells (APCs) increase the expression of cell-surface markers such as CD80/86, MHCII, and CD83, which may influence T-cell polarization to T-helper1 (Th1) [66]. Th1 cells can coordinate cell-mediated immunity, which plays a key role in the control of intracellular pathogens such as *P. salmonis* [67].

However, it has been reported that APCs (MHCII + CD83+) from rainbow trout spleen leukocytes can induce the expression of FOXP3 (Treg polarization-specific transcription factor) in lymphocytes (CD4 + IgM) after induction with IFNγ and *P. salmonis* [68]. This finding suggests a profile associated with immunosuppression or the regulation of homeostasis [68]. A cytokine associated with this immunological profile or process is IL-10 since it has a preserved role in dampening inflammatory responses [59]. Similarly, our data showed that *il-10* was up-regulated in the head kidney and spleen of both EM-90-like and co-infected fish. This could be a mechanism by which the fish avoid harmful exacerbated responses during infection [69], or as proposed by Rozas-Serri et al. [8], it could be an evasion strategy of *P. salmonis* to inactivate the host’s antibacterial response and promote bacterial intracellular survival and replication.

In contrast to the other immune-related biomarkers evaluated, *hepcidin* was detected only in the liver of infected fish (up-regulated in co-infected fish at 14 dpc). Hepcidin is an antimicrobial peptide involved in immunomodulation to resist bacterial infections [70]. Nevertheless, in RTS-11 cells (monocyte/macrophage line of *O. mykiss*), an infection with *P. salmonis* showed a mechanism to ensure pathogen replication and survival inside the cell, inhibiting phagosome-lysosome fusion and preventing the access of hepcidin to vacuoles containing bacteria [71]. Therefore, the up-regulation of *hepcidin* (at 14 dpc) in the liver of co-infected fish could be an attempt by the fish to fight *P. salmonis* or could also be an indicator of disease progression since it was where a higher bacterial load was detected.

Regarding the protein level of imunoglobulins, co-infected fish exhibited a similar detection of total IgM compared to EM-90-like infected fish. This could be due to a greater ratio of EM-90-like to LF-89-like (along the growth curves), which has also been described during in vitro co-cultures [26] and can be supported by the bacterial load results. In general, the production of antibodies seems to be greater against EM-90-like antigens. Even the specific immunoglobulins against *P. salmonis* indicated an interesting cross-reaction by LF-89-like infected fish serum, which detected significantly more EM-90-like antigens. Furthermore, the antigen-specific test detected more of the three antigens in the co-infected fish serum. This result suggested that co-infection with LF-89-like and EM-90-like may promote different virulence determinants toward an antibody response. *P. salmonis* is a facultative intracellular pathogen, and antibody production may not be the best immunological strategy against the development of SRS since the use of resources from the host to produce an adaptive humoral response could be another evasion effort of *P. salmonis* to prevent more robust cell-mediated immunity (e.g., by cytotoxic T cells) [8]. Nevertheless, more research on this topic is necessary, but it could be relevant to consider for the development of more effective vaccines in the future.

The use of an i.p. injection for salmon challenge was the best way to ensure an equal bacterial dose in each fish. However, this is not the natural route of infection, which could affect the results. For instance, it has been reported in fish trials that immersion or cohabitation challenges (using bacteria such as *Aeromonas salmonicida*, *Vibrio proteolyticus*, and *Photobacterium damselae*) are more realistic methods, as they do not bypass the host’s mucosal immunity as an injection does [72]. To help solve this problem, a new infection model to study SRS (by using medaka fish, *Oryzias latipes*) was evaluated. Nevertheless, *P. salmonis* was not able to infect or cause disease in this fish (unpublished results). Thus, in further analysis, a salmon cohabitation challenge would be the most reliable method to mimic a real outbreak of SRS [31]. Moreover, considering our results, a co-infection challenge using both genotypes of *P. salmonis* would improve our understanding of the disease dynamics in the field and the full pathogenic properties of the bacteria.

Fish are naturally surrounded by multiple pathogens in aquatic environments. Thus, concurrent infections are expected to be common. Multiple bacterial or strain infections can have effects on disease dynamics and fish welfare, altering pathogen prevalence and host mortality, and leading to the evolution of more virulent pathogens [30, 73]. In Atlantic salmon, bacterial co-infection studies have been performed using *Moritella viscosa*, the biological agent of typical winter ulcers, a disease that affects salmon farming at low temperatures [74], causing major economic losses in Norway [75]*.* Although *M. viscosa* is considered the main causative agent of this disease, it is often isolated along with *Aliivibrio wodanis* [76–78], which results in more chronic disease and higher mortality rates [74]. Another example is the atypical winter ulcers caused by *Tenacibaculum* spp. and *M. viscosa* [79]. *Tenacibaculum* is also a pathogen that causes skin injuries and is currently among the most relevant fish diseases in Norway, becoming a major cause of fish discards during harvest [11]. Moreover, *Tenacibaculum* is the second cause of death of salmonids (after *P. salmonis*) in Chile [36]. Again, it is necessary to consider bacterial mixed infections in salmon to elucidate the interactions across the pathogen population and their intraspecific genetic diversity [27]. Understanding how these concurrent infections change the disease dynamics and the adverse effects on the host will help to address this host‒interaction and improve the welfare of the fish [80].

There are still knowledge gaps about the mechanism involved in the pathogenicity of *P. salmonis* and its genotypification during the surveillance program on Chilean fish farms. This could shed light on how to develop new effective control measures. Taking co-infection into account may be important for their success since the speciation of *P. salmonis* is ongoing [17], and the impact of this needs to be elucidated.

Our study showed evidence that co-infection with *P. salmonis* LF-89-like and EM-90-like in post-smolt Atlantic salmon affects the within-host SRS disease dynamics. However, it should be noted that these results may depend on the co-culture of the LF-89-like and EM-90-like strains used, as this may impact bacterial growth characteristics and the expression of virulence determinants.

Although *P. salmonis* co-infection modulated immune-related biomarkers in the head kidney, spleen, and liver (up-regulation of cytokines such as *il-1β*, *ifnγ*, *il8*, and *il10*; antimicrobial peptides such as *hepdicin*; PRRs such as *tlr5s*), increased pathological changes in fish (e.g., the formation of ascites, pale nodules in the liver, swollen kidney, and distended ventricle) and decreased survival rate compared to single-infected fish, the potential single-tank effect should be considered in further studies.

Analysis of the bacterial load and immune biomarkers (expression and/or production) suggested a peak time of infection at 14 dpc. In addition, co-infected fish exhibited increased detection of specific IgM against EM-90-like antigens. This finding, coupled with the observed bacterial load in tissue samples, suggested that the EM-90-like isolate might overgrow the LF-89-like isolate during co-infection. Interestingly, even when the LF-89-like isolate did not induce mortality, it is likely that the simultaneous presence of LF-89-like and EM-90-like (and the cross-reactive response) caused a synergistic effect that affected the overall fish health and increased SRS severity. This could be because the response against a multi-genotype infection increases the demand for host resources, but it may also decrease the response to each pathogen separately. Overall, these data contribute to the proposal of the use of a co-infection model for *P. salmonis* to develop more effective control strategies for SRS.

Further field studies are needed to understand the mechanisms that influence host outcomes during *P. salmonis* co-infection. It will provide a deeper understanding of the pathogen population dynamics and their effects on disease development.

## Data Availability

The datasets used during the current study are available from the corresponding author upon reasonable request.
